# Fossils from the Middle Jurassic of China shed light on morphology of Choristopsychidae (Insecta, Mecoptera)

**DOI:** 10.3897/zookeys.318.5226

**Published:** 2013-07-26

**Authors:** Xiao Qiao, Chung Kun Shih, Julian F. Petrulevičius, Ren Dong

**Affiliations:** 1 Key Lab of Insect Evolution & Environmental Change, College of Life Sciences, Capital Normal University, Beijing, 100048, China; 2 Museo de La Plata, División Paleozoología Invertebrados - UNLP, Paseo del Bosque s/n, La Plata (1900), and CONICET, Argentina

**Keywords:** Mecoptera, Choristopsychidae, new genus, new species, Middle Jurassic, China

## Abstract

Choristopsychidae, established by Martynov in 1937 with a single isolated forewing, is a little known extinct family in Mecoptera. Since then, no new members of this enigmatic family have been described. Based on 23 well-preserved specimens with complete body and wings from the Middle Jurassic of northeastern China, we report one new genus and three new species of Choristopsychidae, two new species of the genus *Choristopsyche* Martynov, 1937: *Choristopsyche perfecta* **sp. n.** and *Choristopsyche asticta* **sp. n.**; one new species of *Paristopsyche* **gen. n.**: *Paristopsyche angelineae* **sp. n.**; and re-describe *Choristopsyche tenuinervis* Martynov, 1937. In addition, we emend the diagnoses of Choristopsychidae and *Choristopsyche*. Analyzing the forewing length/width ratios of representative species in Mecoptera, we confirm that choristopsychids have the lowest ratio of forewing length/width, meaning broadest forewings. These findings, the first fossil choristopsychids with well-preserved body structure and the first record of Choristopsychidae in China, shed light on the morphology of these taxa and broaden their distribution from Tajikistan to China, while increasing the diversity of Mesozoic Mecoptera in China.

## Introduction

Choristopsychidae is a rather obscure extinct family in the Order Mecoptera. The family, erected by Martynov in 1937 with an isolated forewing fossil, contains only one species up to date, *Choristopsyche tenuinervis*. Its locality is Shurab II Ditch 63(8), which is in a Pliensbachian terrestrial horizon in the Sulyukta Formation of Tajikistan (Lower Jurassic) (Martynov 1937, Aristov et al. 2009). The family is recognized by a combination of the following characters: forewing broad, ScP long with two long anterior branches; RP and MA with two branches each; MP with five branches; and CuA coalesced with MP basally, strongly bent at about its midpoint (Martynov 1937).

Recently, we collected 23 well-preserved fossils from the Daohugou Village, Ningcheng County, Inner Mongolia, China; Jiulongshan Formation, Middle Jurassic. Herein, based on their different morphological characters, we erect one new genus with one new species and two new species of *Choristopsyche* Martynov, 1937, and re-describe *Choristopsyche tenuinervis* Martynov, 1937, while emending diagnoses of Choristopsychidae Martynov, 1937 and *Choristopsyche* Martynov, 1937.

There are abundant well-preserved fossil insects from Daohugou, including 19 reported orders so far (Ren et al. 2010b). The age of the Daohugou fossil-bearing beds is ca. 164–165 million years ago (Ma) (Bathonian-Callovian boundary interval, the late Middle Jurassic) (Chen et al. 2004).

## Material and methods

This study is based on 23 fossil specimens housed in the fossil insect collection of the Key Laboratory of Insect Evolution & Environmental Changes, College of Life Sciences, Capital Normal University, Beijing, China (CNUB; Dong Ren, Curator).

Photographs of whole specimens were taken with a Nikon D100 digital camera coupled to a Nikkor 105 mm macro lens. The specimens were examined using a Leica MZ12.5 dissecting microscope, and illustrated with the aid of a drawing tube attached to the microscope. Line drawings were made with CoreDRAW X4 graphic software.

**Figure 1.**
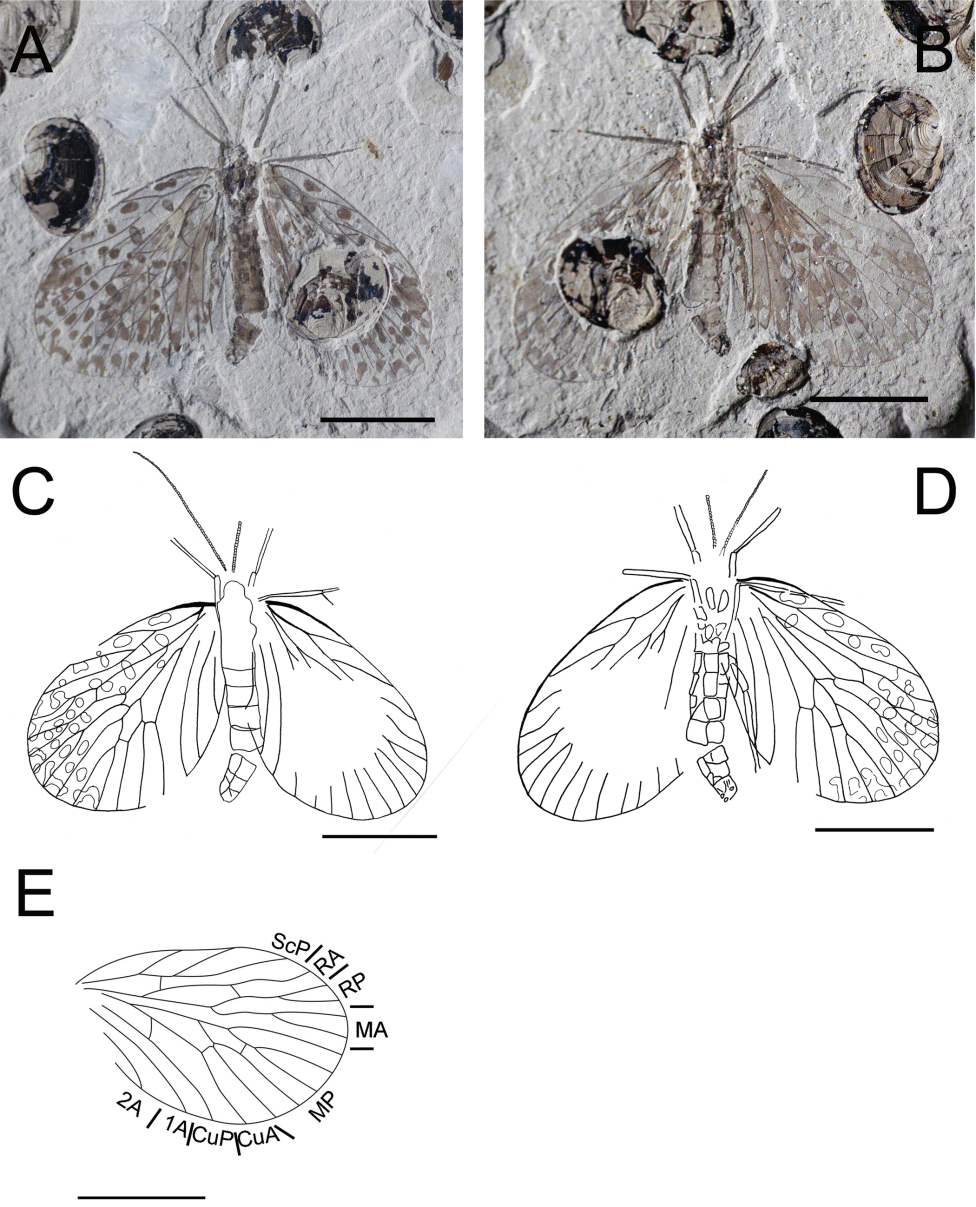
Photographs and line drawings of *Choristopsyche tenuinervis* Martynov, 1937 **A** Photograph of part, no. CNU-MEC-NN2011075p **B** Photograph of counterpart, no. CNU-MEC-NN2011075c **C** Line drawing of whole specimen of part, no. CNU-MEC-NN2011075p **D** Line drawing of whole specimen of counterpart, no. CNU-MEC-NN2011075c **E** Line drawing of forewing of part, no. CNU-MEC-NN2011075p. Scale bars represent 5 mm.

The wing venation nomenclature used in this paper is based on the interpretations and system proposed by Novokshonov (2002) with some revisions, Corresponding abbreviations are: ScP, posterior Subcosta; RA, anterior Radius; RP, posterior Radius; MA, anterior Media; MP, posterior Media; CuA, anterior Cubitus; CuP, posterior Cubitus; 1A, the first anal vein; 2A, the second anal vein (Fig. 1E). The length of the wing is measured by the straight-line distance from the wing base to apex, and the width of the wing, the straight-line distance from the wing anterior margin to posterior margin at its broadest point.

## Systematic palaeontology

### Order Mecoptera Packard, 1886

#### Family Choristopsychidae

Martynov, 1937

http://species-id.net/wiki/Choristopsychidae

##### Emended diagnosis.

Forewing broad oval or subtriangular, field between C and ScP comparatively broad; ScP well developed and forked twice, forming three long branches; RA unforked, one crossvein between ScP and RA and between RA and RP; RP and MA both with two branches; MP with five branches, and the MP_4+5_ forking basal to the MP_2+3_ forking; MP and CuA merged at the base; CuA strongly bent at its mid point; an oblique crossvein between CuA and CuP; a curved crossvein between the midpoint of CuA and MP_5_; CuP, 1A and 2A almost parallel. Hind wing, similar in shape to the forewing but slightly smaller, ScP short, forked twice, the second bifurcation coalesces with RA for a short distance; RP and MA both with two branches; MP with five branches, the stem of MP_4+5_ forked earlier than that of forewing, and with a crossvein to CuA; CuA almost straight. Head, oviform with big and oval compound eyes; antennae long and filiform; small chewing mouthpart. Thorax: prothorax smaller than mesothorax and metathorax. Legs: long and slender, all legs nearly of the same shape, but hind legs longer than fore legs and mid legs, and femora wider than tibia, and tibia longer than femora. Abdomen slender, tapering apically, about eleven segments and the female terminal segment with cercus.

#### Genus Choristopsyche

Martynov, 1937

http://species-id.net/wiki/Choristopsyche

##### Type species.

*Choristopsyche tenuinervis* Martynov, 1937 (Lower Jurassic of Tajikistan)

##### Emended diagnosis.

Forewing, the separation of RP+MA from RA distal to the separation of MP from CuA.

##### Included species.

Type species (*Choristopsyche tenuinervis* Martynov, 1937), *Choristopsyche perfecta* sp. n. and *Choristopsyche asticta* sp. n.

#### Choristopsyche tenuinervis

Martynov, 1937

http://species-id.net/wiki/Choristopsyche_tenuinervis

Figs 1 , 2

##### Emended diagnosis.

Forewing, RP+MA forking distal to MP forking.

##### Description of new material.

CNU-MEC-NN2011075p/c (Fig. 1), a well preserved specimen with part and counterpart in dorsal view, with almost complete forewings, but partially preserved hind wings and body, and forewings overlapping hind wings. The terminus of abdomen is missing, sex unknown. Wings: Left forewing, length 11.0 mm, width 6.7 mm, broadly oval, RP forking distal to MA forking; MP_2+3_ forking basal to the forking of MA, and the stem of MP_3_ about twice as long as the stem of MP_2+3_; with one crossvein between RA and RP, and between MP_2+3_ and MP_4_; CuP, 1A, 2A single. Right forewing is similar to left forewing, but parts missing. Hind wings, smaller than forewings, overlapped by forewings, the venation visible but unclear. There are many spots on all four wings, symmetric between left and right wings.

In addition, there are nine new materials with analogous wing venation to that of specimen CNU-MEC-NN2011075p/c. They are listed as follows.

**Figure 2.**
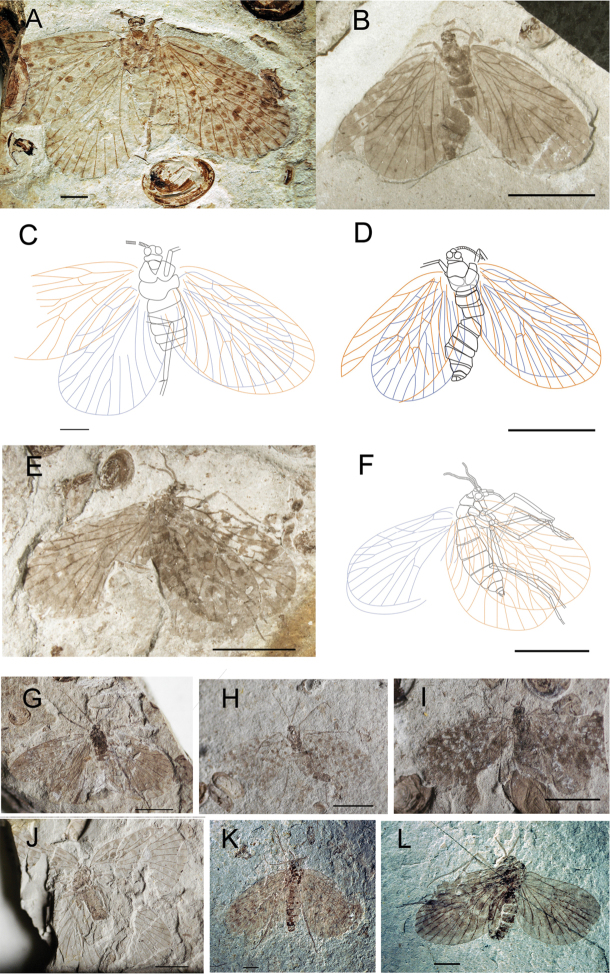
Photographs and line drawings of *Choristopsyche tenuinervis* Martynov, 1937 **A** Photograph of no. CNU-MEC-NN2011080 **B** Photograph of no. CNU-MEC-NN2009317 **C** Line drawing of no. CNU-MEC-NN2011080**D** Line drawing of no. CNU-MEC-NN2009317 **E** Photograph of no. CNU-MEC-NN2009414 **F** Line drawing of no. CNU-MEC-NN2009414 **G** Photograph of no. CNU-MEC-NN2009318 **H** Photograph of no. CNU-MEC-NN2011070 **I** Photograph of no. CNU-MEC-NN2011071 **J** Photograph of no. CNU-MEC-NN2009383 **K** Photograph of no. CNU-MEC-NN2011083 **L** Photograph of no. CNU-MEC-NN2011085. Scale bars of**A, C, K, L** represent 2 mm. Scale bars of **B, D, E, F, G, H, I, J** represent 5 mm.

CNU-MEC-NN2011080 (Figs 2A, C), a well preserved specimen with clear wings, but parts of body, and the right forewing overlapping the right hind wing. Sex unknown. Wing: Right forewing, length 11.8 mm, width 6.7 mm, RP forking distal to the forking of MA; MP_2+3_ forking at about the same level as the forking of MA; the stem of MP_3_ about twice as long as the stem of MP_2+3_; with one crossvein between MP_2+3_ and MP_4_; CuP, 1A, 2A single. Left forewing is similar to right forewing, but the apex of the wing absent. Hind wings, length at about 10.1 mm, width 6.2 mm, similar to forewings but smaller. CNU-MEC-NN2009317 (Figs 2B, D), an almost complete specimen, female, with forewings overlapping hind wings, and nearly complete body, but legs absent in dorsal view. Wings: right forewing, length at about 9.5 mm, width 4.9 mm; RP forking distal to the forking of MA; MP_2+3_ forking at about the same level to the forking of MA; the stem of MP_3_ as long as the stem of MP_2+3_; CuP, 1A, 2A single. Left forewing is similar to right forewing. Hind wings, similar to forewings, but smaller; CuA almost straight. CNU-MEC-NN2009414 (Figs 2E, F), an almost complete preserved specimen in lateral view, female, with complete body and forewings, and right forewing overlapped with body and parts of left forewing, and right hind wing overlapped with left hind wing. The mouthparts are missing, the maxillary palpus with five segments visible. Abdomen: tapering apically, with eleven segments, and a pair of cercus can be visible, female. Wings: Left forewing, length at about 10.1 mm, width 6.4 mm, broadly oval, RP forking basal to the forking of MA; MP_2+3_ forking significantly basal to the forking of MA; the stem of MP_3_ about three times as long as the stem of MP_2+3_; with one crossvein between RA and RP, and between MA and MP_1_; CuP, 1A, 2A single. Right forewing is similar to left forewing, but there are crossveins between ScP and RA, RA and RP, MA and MP_1_. Hind wing: similar to forewing but smaller, RA straight, with one crossvein to RP. CNU-MEC-NN2009318 (Fig. 2G), a partially preserved specimen in dorsal view, with parts of forewings, hind wings and body, but the filiform antennae and venation visible, and the forewings overlapping the hind wings. CNU-MEC-NN2011070 (Fig. 2H), an almost completely preserved specimen in ventral view, with almost complete body and four wings, and hind wings overlapped with forewings. CNU-MEC-NN2011071 (Fig. 2I), a partially preserved specimen in dorsal view, with parts of body and forewings, but left hind wing is missing, and right hind wing is obscure. CNU-MEC-NN2009383 (Fig. 2J), a partially preserved specimen with four outspread wings and parts of body. CNU-MEC-NN2011083 (Fig. 2K), a specimen in dorsal view, female, with almost complete body but some legs not visible due to coverage by wings, and forewings overlapping hind wings. CNU-MEC-NN2011085 (Fig. 2L), a comparatively complete specimen in lateral view, male, and forewings overlapping hind wings; chewing mouthparts visible; Abdomen almost completely-preserved, the posterior six segments can be seen clearly, and abdomen bent at six and seven segment, but the posterior segments faint below the left forewing, the scorpion-like terminal visible.

##### New material.

CNU-MEC-NN2011075p/c, CNU-MEC-NN2011080, CNU-MEC-NN2009317, CNU-MEC-NN2009414, CNU-MEC-NN2009318, CNU-MEC-NN2011070, CNU-MEC-NN2011071, CNU-MEC-NN2009383, CNU-MEC-NN2011083, CNU-MEC-NN2011085, deposited in CNUB.

##### Type locality and horizon.

Daohugou Village, Ningcheng County, Inner Mongolia, China, Jiulongshan Formation, Middle Jurassic (Bathonian-Callovian boundary interval, ca 164–165 Ma).

##### Remarks.

These ten specimens exhibit differences in the characters of “RP forking vs. MA forking”, “MP_2+3_ forking vs. MA forking” and “Length ratio of the stem of MP_3_ and the stem of MP_2+3_”, which are considered as intraspecific variations.

#### Choristopsyche perfecta sp. n.

urn:lsid:zoobank.org:act:FA6A6E51-B3BD-459E-AED4-021539147BA3

http://species-id.net/wiki/Choristopsyche_perfecta

Fig. 3

##### Diagnosis.

Forewing, RP+MA forking almost at the same level to MP forking.

##### Description.

**Figure 3.**
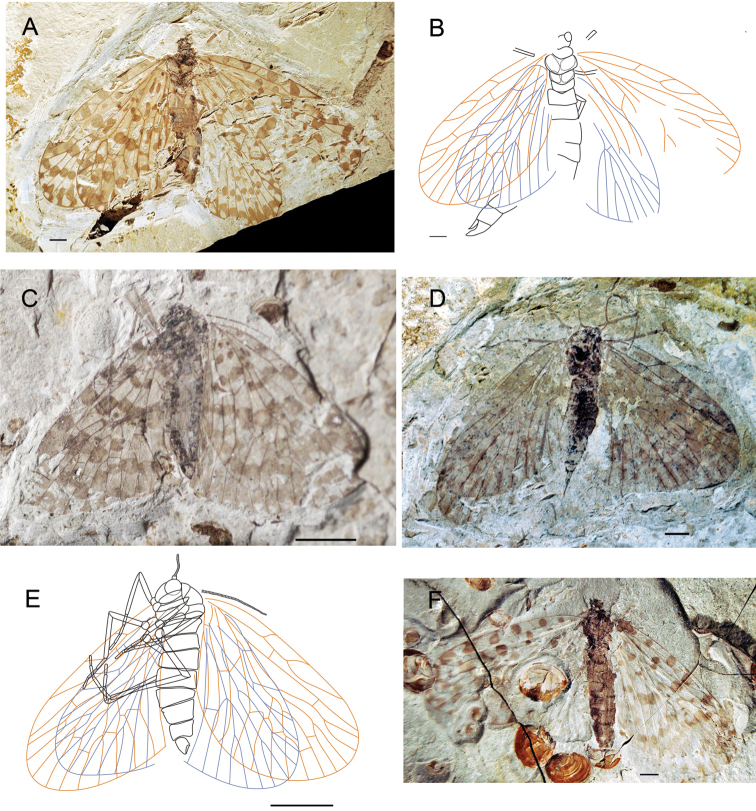
Photographs and line drawings of *Choristopsyche perfecta* sp. n. **A** Photograph of holotype, no. CNU-MEC-NN2011082 **B** Line drawing of holotype, no. CNU-MEC-NN2011082 **C** Photograph of paratype, no. CNU-MEC-NN2009352 **D** Photograph of paratype, no. CNU-MEC-NN2011079 **E** Line drawing of paratype, no. CNU-MEC-NN2009352 **F** Photograph of paratype, no. CNU-MEC-NN2011084. Scale bars of **A, B, D, F** represent 2 mm; scale bars of **C, E** represent 5 mm.

HolotypeCNU-MEC-NN2011082 (Figs 3A, B), an almost complete preserved specimen, maybe male, with four outspread wings, but with partially preserved body. Head is partially preserved, only with one compound eye visible. Thorax: prothorax smaller than mesothorax and metathorax in ventral view. Legs: some parts of fore legs and left hind leg visible. Abdomen: tapering apically, with six segments visible, but the terminal visible, maybe male. Wings: Right forewing, length 22.2 mm, width at about 11.4 mm, RP forking distal to the forking of MA; MP_2+3_ forking distal to the forking of MA; the stem of MP_3_ about twice as long as the stem of MP_2+3_; with one crossvein between CuP and 1A; CuP, 1A single. Left forewing is partially preserved, similar to right forewing. Hind wings, length 18.4 mm, width at about 10.3 mm, similar to forewings but smaller than forewings, and left hind wing partially preserved. There are many spots on all four wings, symmetric between left and right wings.

Paratypes: CNU-MEC-NN2009352 (Figs 3C, E), an almost complete preserved specimen, with clear wing venation and structure of body in side pressure, and forewings partially overlapped with hind wings, sex unknown. Wings: Left forewing, length 18.8 mm, width 10.0 mm, RP forking slightly distal to the forking of MA; MP_2+3_ forking at about the same level to the forking of MA; the stem of MP_3_ about twice as long as the stem of MP_2+3_; with one crossvein between MP_1_ and MA_4_, MP_1_ and MP_2_, MP_2+3_ and MP_4_; CuP, 1A, 2A, 3A single, and one crossvein between CuP and 1A. Right forewing is similar to left forewing. Hind wing, similar to forewing, but smaller. CNU-MEC-NN2011079 (Fig. 3D), female, a well-preserved specimen with complete body, and the terminal of abdomen visible, but the end of legs absent, and forewings overlapping hind wings, but parts of wings missing, RP forking distal to the forking of MA; Abdomen: tapering apically, with eleven visible segments, the tenth and eleventh segments smaller, and the eleventh segment with cerci visible. CNU-MEC-NN2011084 (Fig. 3F), a specimen with legs absent, and hind wings overlapped by forewings in dorsal view, sex unknown. Forewing, length 19.4 mm, width 10.0 mm, RP forking at the same level to the forking of MA; MP_2+3_ forking basal to the forking of MA; the stem of MP_3_ about twice as long as the stem of MP_2+3_.

##### Material.

Holotype CNU-MEC-NN2011082, Paratypes CNU-MEC-NN2009352, CNU-MEC-NN2011079, CNU-MEC-NN2011084, deposited in CNUB.

##### Type locality and horizon.

Daohugou Village, Ningcheng County, Inner Mongolia, China, Jiulongshan Formation, Middle Jurassic (Bathonian-Callovian boundary interval, ca 164–165 Ma).

##### Etymology.

The name is derived from the Latin word of *perfectus*, meaning “complete”.

##### Remarks.

These four specimens exhibit differences in the character of “RP forking vs. MA forking”, “MP_2+3_ forking vs. MA forking”, which is considered as intraspecific variations.

#### Choristopsyche asticta sp. n.

urn:lsid:zoobank.org:act:31DA887F-2987-4DAD-9520-5776FA0CD5CF

http://species-id.net/wiki/Choristopsyche_asticta

Fig. 4

##### Diagnosis.

Forewing, RP+MA forking basal to MP forking.

##### Description.

**Figure 4.**
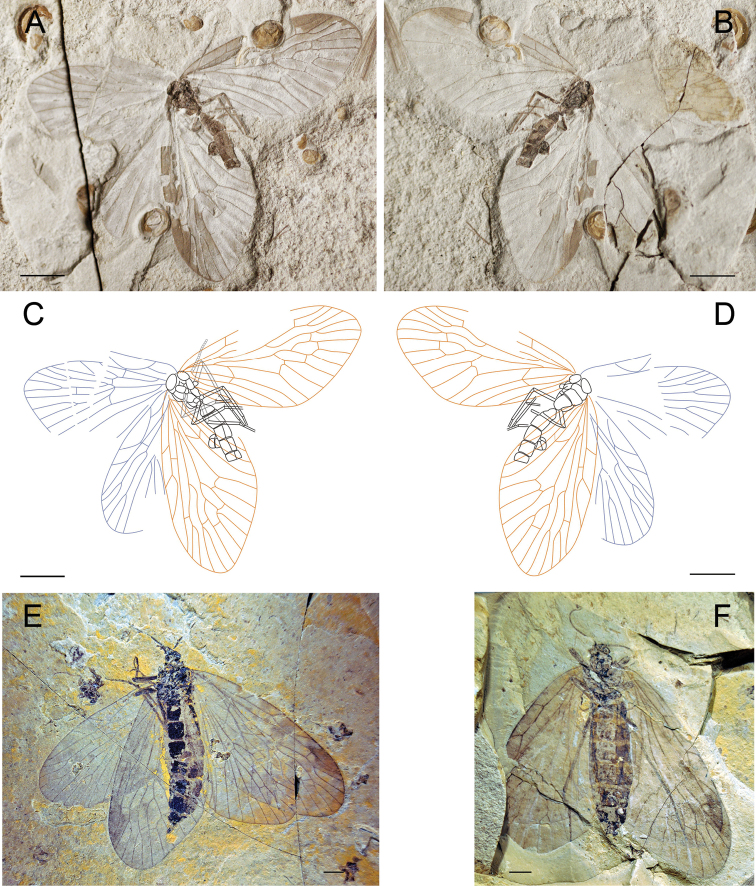
Photographs and line drawings of *Choristopsyche asticta* sp. n. **A** Photograph of part of holotype, no. CNU-MEC-NN2009394p **B** Photograph of counterpart of holotype, no. CNU-MEC-NN2009394c **C** Line drawing of part of holotype, no. CNU-MEC-NN2009394p **D** Line drawing of counterpart of holotype, no. CNU-MEC-NN2009394c **E** Photograph of paratype, no. CNU-MEC-NN2011081 **F** Photograph of paratype, no. CNU-MEC-NN2011086. Scale bars of **A–D** represent 5 mm. Scale bars of **E–F** represent 2 mm.

Holotype, CNU-MEC-NN2009394p/c (Figs 4A–D), an almost complete specimen, female, with well-preserved four outspread wings. Head: compound eyes are big and oval in ventral view, but mouthparts invisible and antenna partially preserved. Thorax: prothorax smaller than mesothorax and metathorax, visible in ventral view. Legs: all legs nearly the same shape and nearly completely preserved, long and slender in ventral view. Abdomen: slender and elongate, tapering apically, with eight visible segments. Wings: four wings are elongated and broad, with rounded apical margin. Forewings: Right forewing, length 20.7 mm, width 10.2 mm, almost triangular, dark color between C and RA; RP forking basal to the forking of MA; MP_2+3_ forking at about the same level to the forking of MA; the stem of MP_3_ about twice as long as the stem of MP_2+3_; the stem of MA strongly bent posteriorly; and the stem of MP_2+3_ strongly bent at its basal one third part; with one crossvein between MA and MP_1_, MP_1_ and MP_2_, and between MP_2+3_ and MP_4_; and one oblique crossvein between the base of CuA and CuP, and at the point one oblique crossvein between CuP and 1A, CuP, 1A, 2A single. Left forewing is similar to right forewing. Hind wings: similar to forewing, but slightly smaller, length at about 16.8 mm, width at about 10.0 mm; Right hind wing, with one crossvein between RA and RP+MA. Left hind wing is similar to right hind wing. No spots on entire wings.

Paratypes: CNU-MEC-NN2011081 (Fig. 4E), female, a well-preserved specimen, with complete body and wings; Head, oval with two big compound eyes, filiform antenna and chewing mouthparts; Abdomen, slender and elongate in lateral view, tapering apically and complete preserved, length at about 13.4 mm; Wings, forewings overlapping some parts of hind wings, and with clear venation; Forewing, length 19.8 mm, width 8.4 mm, RP forking basal to the forking of MA; MP_2+3_ forking at the same level to the forking of MA; the stem of MP_3_ about twice as long as the stem of MP_2+3_. Hind wing, length at about 16.6 mm, width 8.1 mm. CNU-MEC-NN2011086 (Fig. 4F), female, an fairly well-preserved specimen in dorsal view, forewings overlapping hind wings, with almost complete body; Forewing, length 20.8 mm, width at about 9.4 mm; RP forking basal to the forking of MA; MP_2+3_ forking at the same level to the forking of MA; the stem of MP_3_ about twice as long as the stem of MP_2+3_. Hind wing, length 17.3 mm, width at about 9.0 mm.

##### Material.

Holotype CNU-MEC-NN2009394p/c, Paratypes CNU-MEC-NN2011081, CNU-MEC-NN2011086, deposited in CNUB.

##### Type locality and horizon.

Daohugou Village, Ningcheng County, Inner Mongolia, China, Jiulongshan Formation, Middle Jurassic (Bathonian-Callovian boundary interval, ca 164–165 Ma).

##### Etymology.

The name is derived from the Latin word of *astictus*, meaning “no spots”.

#### Paristopsyche gen. n.

urn:lsid:zoobank.org:act:B960BA0B-429E-4BCE-96D5-D59BDB7CC263

http://species-id.net/wiki/Paristopsyche

##### Type species.

*Paristopsyche angelineae* sp. n..

##### Diagnosis.

Forewing, the separation of RP+MA from RA at about the same level as the separation of MP from CuA.

##### Included species.

Type species: *Paristopsyche angelineae* sp. n..

##### Etymology.

The name is derived from the Greek word of *paris-*, meaning “equal”, and *psyche*, from the Greek, meaning “soul” or “mind”. The gender is feminine.

#### Paristopsyche angelineae sp. n.

urn:lsid:zoobank.org:act:6C1AA33E-D1B1-4D52-9B7F-240CFA9FB1EB

http://species-id.net/wiki/Paristopsyche_angelineae

Figs 5 , 6

##### Diagnosis.

Forewing, RP+MA forking distal to MP forking.

##### Description.

**Figure 5.**
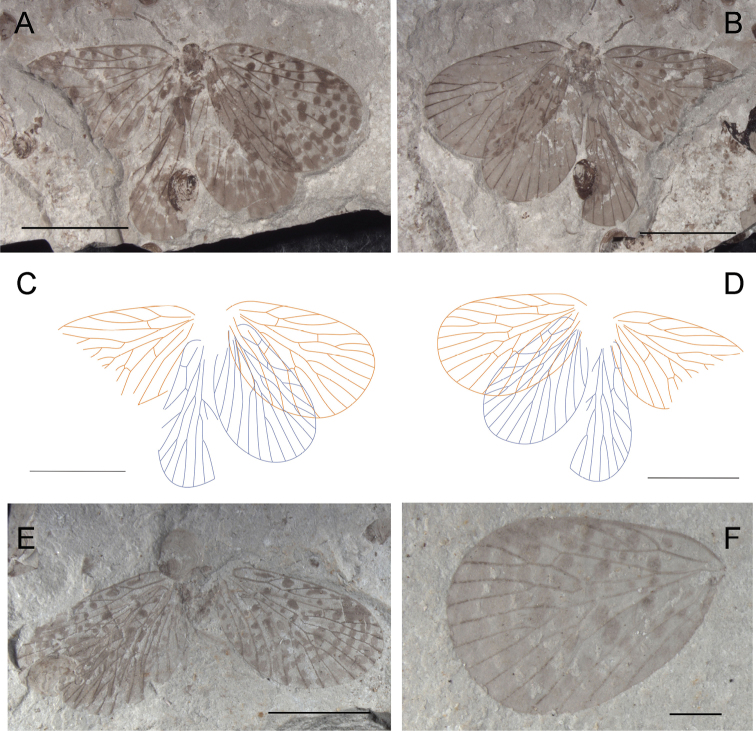
Photographs and line drawings of *Paristopsyche angelineae* gen. et sp. n. **A** Photograph of part of holotype, no. CNU-MEC-NN2011076p **B** Photograph of counterpart of holotype, no. CNU-MEC-NN2011076c **C** Line drawing of part of holotype, no. CNU-MEC-NN2011076p **D** Line drawing of counterpart of holotype, no. CNU-MEC-NN2011076c **E** Photograph of paratype, no. CNU-MEC-NN2009319 **F** Photograph of paratype, no. CNU-MEC-NN2011074. Scale bars of **A–E** represent 5 mm. Scale bar of **F** represents 1 mm.

Holotype, CNU-MEC-NN2011076p/c (Figs 5A–D), a well preserved specimen in dorsal view, with outspread clear wings, but parts of body visible. Some segments of head, thorax, legs visible, but faint. Wings: Right forewing, length 8.4 mm, width 5.5 mm, broadly oval with clear venation, RP forking distal to MA forking; MP_2+3_ forking at about the same level of the forking of MA; the stem of MP_3_ about three times as long as the stem of MP_2+3_; with one crossvein between MA and MP_1+2+3_, and between MP_2+3_ and MP_4_. Left forewing is similar to right forewing, but the apex of the wing absent. Right hind wing, length at about 7.5 mm, width 4.6 mm, similar to forewing, but smaller, and overlapping with forewing partially. Left hind wing is similar to left hind wing, but partly folded as preserved. There are many spots on all four wings, symmetric between left and right wings.

**Figure 6.**
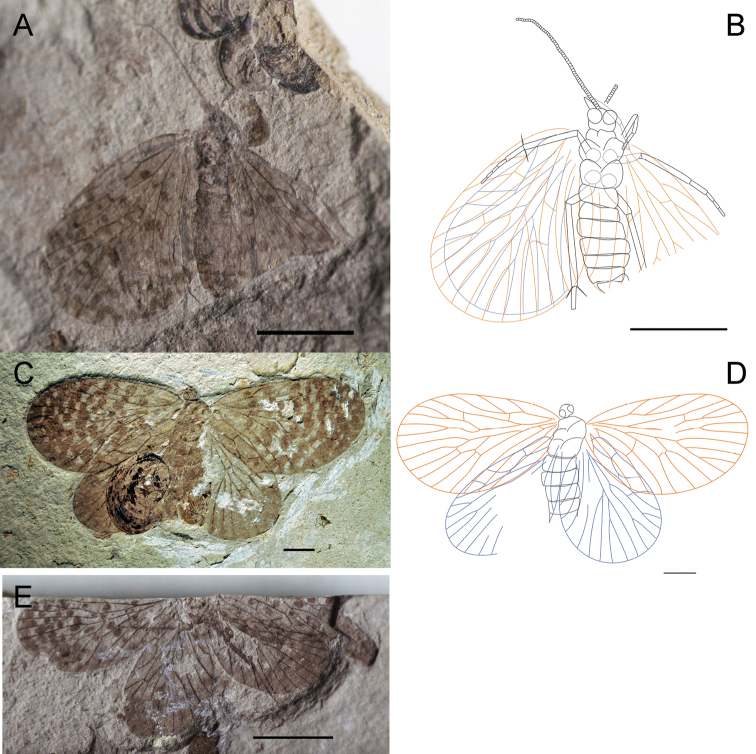
**A–B** Photographs and line drawings ofparatypesof *Paristopsyche angelineae* sp. n. **A** Photograph of paratype, no. CNU-MEC-NN2011069 **B** Line drawing of paratype, no. CNU-MEC-NN2011069 **C** Photograph of paratype, no. CNU-MEC-NN2011078 **D** Line drawing of paratype, no. CNU-MEC-NN2011078 **E** Photograph of paratype, no. CNU-MEC-NN2011077. Scale bars of **A, B, E** represent 5 mm. Scale bars of **C-D** represent 2 mm.

Paratypes: CNU-MEC-NN2009319 (Fig. 5E), a partially preserved specimen with forewings and one hind wing, but body absent; forewing, length 9.0 mm, width 5.4 mm, RP forking distal to MA forking; MP_2+3_ forking at about the same level of the forking of MA; the stem of MP_3_ about three times as long as the stem of MP_2+3_. CNU-MEC-NN2011074 (Fig. 5F), a specimen with one complete and clear forewing, length 7.4 mm, width 5.2 mm, RP forking distal to MA forking; MP_2+3_ forking at about the same level of the forking of MA; the stem of MP_3_ about three times as long as the stem of MP_2+3_. CNU-MEC-NN2011069 (Figs 6A, B), a partially preserved specimen, with complete left wings and most of body except for the terminalia, but incomplete right wings in dorsal view. Wings: right forewing, length 11.2 mm, width 7.5 mm, broadly oval, field between C and RA wide; RP forking distal to MA forking; MP_2+3_ forking distal to the forking of MA; the stem of MP_3_ about twice as long as the stem of MP_2+3_; CuP, 1A, 2A, single. Right hind wing is similar to forewing, but slightly smaller. Left wings partially preserved. CNU-MEC-NN2011078 (Figs 6C, D), an almost preserved specimen in dorsal view, with outspread clear wings, but parts of body absent. Wings: Left forewing, length 10.7 mm, width 6.4 mm, RP forking at the same level of the forking of MA; MP_2+3_ forking basal to the forking of MA; the stem of MP_3_ about four times as long as the stem of MP_2+3_; with one crossvein between MP_3_ and MP_4_, and between 1A and 2A; CuP, 1A, 2A single. Right forewing, similar to left forewing, but individual asymmetry is shown by right wing having MP_3_ with two branches, not the typical one branch in the left wing. Hind wings, length 8.2 mm, width 6.2 mm, similar to forewings but smaller, and partially preserved. CNU-MEC-NN2011077 (Fig. 6E), a partially preserved specimen with complete hind wings but parts of forewings and body; Forewing, length at about 10.3 mm, RP forking at the same level of the forking of MA; MP_2+3_ forking basal to the forking of MA; the stem of MP_3_ about three times as long as the stem of MP_2+3_; Hind wing, length at about 7.9 mm, width 5.0 mm.

##### Material.

Holotype CNU-MEC-NN2011076p/c, Paratypes CNU-MEC-NN2011078, CNU-MEC-NN2011077, CNU-MEC-NN2011069, CNU-MEC-NN2009319, CNU-MEC-NN2011074, deposited in CNUB.

##### Type locality and horizon.

Daohugou Village, Ningcheng County, Inner Mongolia, China, Jiulongshan Formation, Middle Jurassic (Bathonian-Callovian boundary interval, ca 164–165 Ma).

##### Etymology.

The specific name is dedicated to Ms. Janet Angeline for her professionalism, dedication and accomplishments in her field and providing inspiration and support to CKS’s palaeontology studies.

##### Remarks.

These six specimens exhibit differences in the characters of “RP forking vs. MA forking”, “MP_2+3_ forking vs. MA forking” and “Length ratio of the stem of MP_3_ and the stem of MP_2+3_”, which are considered as intraspecific variations.

## Discussion

Willmann (1989) synonymized Choristopsychidae with Agetoparnopidae Carpenter, 1930 because of forewing venation. Novokshonov (2002) synonymized Choristopsychidae and Agetoparnopidae with Permochoristidae Tillyard, 1917 also because of wing venation of the forewing. Based on our new findings about the characters of Choristopsychidae and the reported family of Agetoparnopidae by Carpenter (1930) and Permochoristidae by Tillyard (1917), there are significant differences between Choristopsychidae, Agetopanorpidae and Permochoristidae. For example, the former has broad oval wings (vs. long and narrow), MA vein with 2 branches (vs. 3 branches), MP vein with 5 branches (vs. 6 branches), CuA strongly bent at its mid point (vs. CuA without bending) etc. Therefore, we consider that it is justifiable for Martynov in setting up the family of Choristopsychidae.

Based on studies of our specimens and the reported species of Choristopsychidae, we found the wing venation of this family is comparatively stable, that is, the numbers of branches of ScP, RP, MA, MP have almost no changes in these specimens. However we observed significant differences regarding the relative positions between bifurcation points of two different veins, especially the origination locations of the main veins. In the literature of Mecoptera taxonomy, these characters have been used as diagnostic characters for genera or species, such as in Orthophlebiidae, Aneuretopsychidae and so on (Hong and Zhang 2007, Qiao et al. 2012a, 2012b, Ren et al. 2011). Therefore, we contend the character of “the separation of RP+MA from RA distal, basal or at the same level to the separation of MP from CuA” is the generic diagnostic character for Choristopsychidae; and the character of “the relative positions (basal or distal) between the forking of RP+MA and the forking of MP” is the specific diagnostic character. On the other hand, to avoid over classification of species, we also consider the characters of “RP forking vs. MA forking”, “MP_2+3_ forking vs. MA forking” and “Length ratio of the stem of MP_3_ and the stem of MP_2+3_” are intraspecific variations.

**Figure 7.**
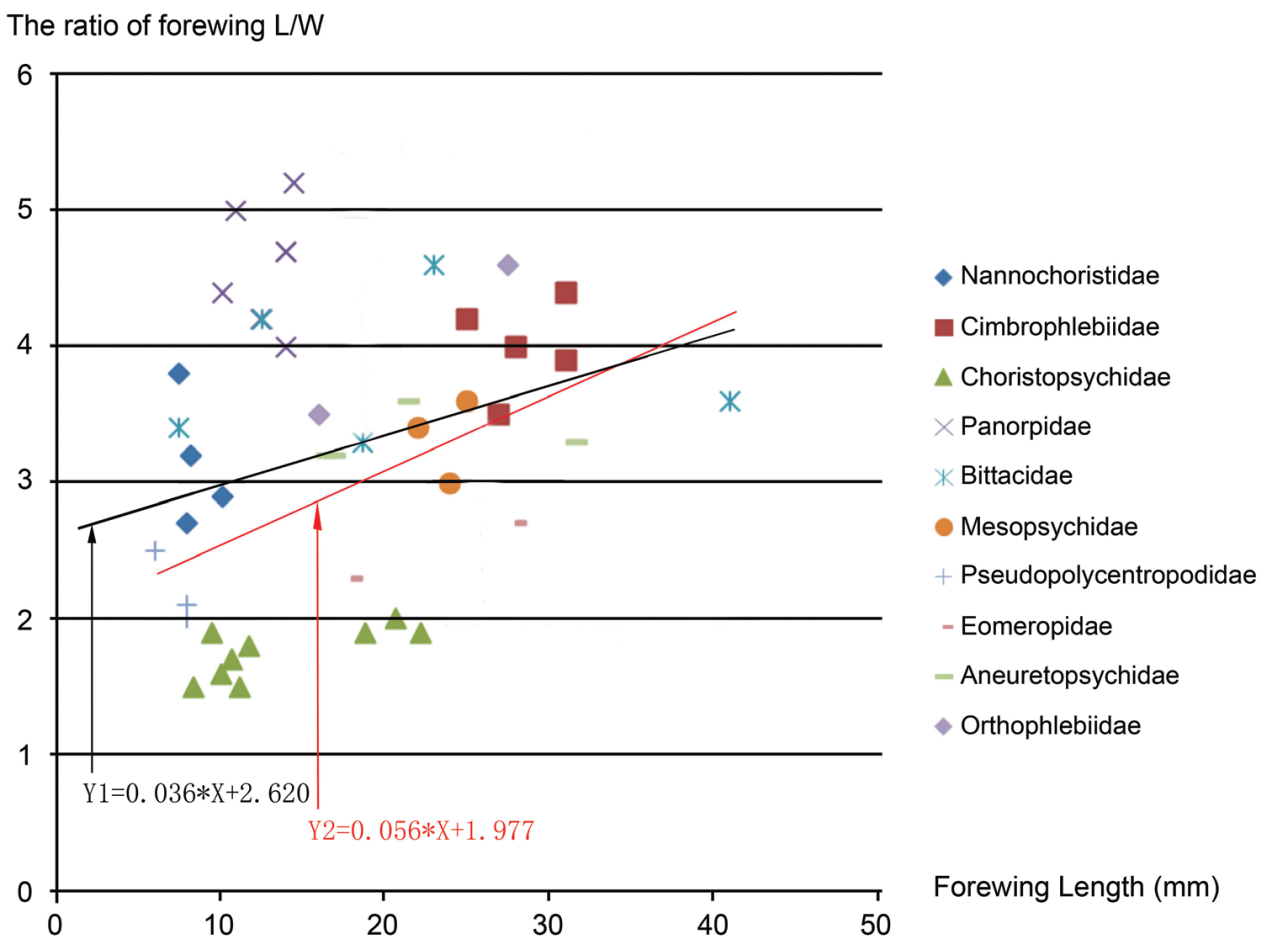
The ratio of forewing length (L)/width (W) vs. forewing length (in mm) of ten representative Families of Mecoptera. For all data points, the linear regression trend line is represented by Y1 = 0.036*X + 2.620. Excluding the data of Panorpidae, the linear regression trend line is Y2 = 0.056*X + 1.977.

Choristopsychids have relatively broad wings, either oval or subtriangular-shaped, with length/width ratio from 1.5 to 2.0, in contrast to long and narrow wings of most mecopterans. To compare relative forewing broadness for representative mecopterans, we summarize the data of forewing length, width and ratio of wing length/width in Table 1 and plot the ratio of wing length/width vs. wing length (in mm) in Fig. 7. The data and Fig. 7 indicate the family of Choristopsychidae has the lowest ratio, meaning broadest forewings among mecopterans. In addition, the data and Fig. 7 seem to show a general trend that for representative specimens of these families, the larger the body size, the narrower the forewings (comparatively higher ratio). The linear regression trend line is represented by Y1 = 0.036*X + 2.620. For example, the family of Cimbrophlebiidae have large body size, with forewing length from 25 mm to 30 mm, and high ratio, with L/W ratio from 3.5 to 4.4 (Bruce 2009, Yang et al. 2012b). It is also noted that Panorpidae have unusually high ratio (more slender), with L/W ratio from 4 to 5, for their relatively small body size, with forewing length from 10 mm to 14 mm (Fu and Hua 2009, Zhou et al. 1993). If we exclude the data of Panorpidae, the linear regression trend line is Y2 = 0.056*X + 1.977. The higher value of slope indicates clearer trend that the larger the body size, the narrower the forewings for all these families of Mecoptera excluding Panorpidae.

**Table 1.** Data of forewing length, width and length/width ratio of representatives of ten Families in Mecoptera.

**Family**	**Genus**	**Species**	**No. of fossil**	**Length of forewing (mm)**	**Width of forewing (mm)**	**Ratio of length/width**
Aneuretopsychidae Rasnitsyn & Kozlov, 1990	*Jeholopsyche* Ren, Shih & Labandeira, 2011	*Jeholopsyche liaoningensis* Ren, Shih & Labandeira, 2011	CNU-M-LB2005002	21.5	6	3.6
		*Jeholopsyche completa* Qiao, Shih & Ren, 2012	CNU-MEC-LB2011062	16.5	5.2	3.2
		*Jeholopsyche bella* Qiao, Shih & Ren, 2012	CNU-MEC-LB2011063	17	5.4	3.2
		*Jeholopsyche maxima* Qiao, Shih & Ren, 2012	CNU-MEC-LB2011064	31.7	8.5	3.7
Orthophlebiidae Handlirsch, 1906	*Orthophlebia* Westwood, 1845	*Orthophlebia liaoningensis* Ren, 1997	LB95055	16	4.6	3.5
		*Orthophlebia nervulosa* Qiao, Shih & Ren, 2012	CNU-MEC-NN2011060	27.5	6.0	4.6
Eomeropidae Cockerell, 1909	*Tsuchingothauma* Ren & Shih, 2005	*Tsuchingothauma shihi* Ren & Shih, 2005	M-NN200401	28	10.5	2.7
	*Typhothauma* Ren & Shih, 2005	*Typhothauma yixianensis* Ren & Shih, 2005	M-LB200401	18	8	2.3
Pseudopolycentropodidae Handlirsch, 1925	*Pseudopolycentropus* Handlirsch, 1906	*Pseudopolycentropus janeannae* Ren, Shih & Labandeira, 2010	CNU-M-NN2005001	8	4	2
		*Pseudopolycentropus novokshonovi* Ren, Shih & Labandeira, 2010	CNU-M-NN2005002	8	3.9	2.1
	*Sinopolycentropus* Shih, Yang & Labandeira, 2011	*Sinopolycentropus rasnitsyni* Shih, Yang & Labandeira, 2011	CNU-MEC-NN2010044	6.1	2.4	2.5
Cimbrophlebiidae Willmann, 1977	*Cimbrophlebia* Willmann, 1977	*Cimbrophlebia flabelliformis* Bruce, 2009	UCCIPR L-18 F-763	28	7	4
		*Cimbrophlebia leahyi* Bruce, 2009	TRUIPR L-018 F-1161	31	8	3.9
		*Cimbrophlebia brooksi* Bruce, 2009	SR062005	31	7	4.4
		*Cimbrophlebia westae* Bruce, 2009	SRUI099600	25	6	4.2
	*Perfecticimbrophlebia* Yang, Shih & Ren, 2012	*Perfecticimbrophlebia laetus* Yang, Shih & Ren, 2012	CNU-M-NN2010004	26.9	7.6	3.5
Nannochoristidae Tillyard, 1917	*Protochoristella* Sun, Ren & Shih, 2007	*Protochoristella polyneura* Sun, Ren & Shih, 2007	CNU-M-NN2006049	7.5	2	3.8
		*Protochoristella formosa* Sun, Ren & Shih, 2007	CNU-M-NN2006006	8	3	2.7
	*Itaphlebia* Sukatsheva, 1985	*Itaphlebia exquisita* Liu, Zhao & Ren, 2010	CNU-MEC-NN2009145	10.2	3.5	2.9
		*Itaphlebia laeta* Liu, Zhao & Ren, 2010	CNU-MEC-NN2009311	8.2	2.6	3.2
Mesopsychidae Tillyard, 1917	*Lichnomesopsyche* Ren, Labandeira & Shih, 2010	*Lichnomesopsyche gloriae* Ren, Labandeira & Shih, 2010	CNU-M-NN2005020	25	7	3.6
		*Lichnomesopsyche daohugouensis* Ren, Labandeira & Shih, 2010	CNU-M-NN2005022	22	6.5	3.4
	*Vitimopsyche* Novokshonov & Sukatasheva, 2001	*Vitimopsyche kozlovi* Ren, Labandeira & Shih, 2010	CNU-M-HP2005001	24	8	3
Bittacidae Handlirsch, 1906	*Exilibittacus* Yang, Ren & Shih, 2012	*Exilibittacus lii* Yang, Ren & Shih, 2012	CNU-M-NN2010001	7.5	2.2	3.4
	*Preanabittacus* Novokshonov, 1993	*Preanabittacus validus* Yang, Ren & Shih, 2012	CNU-MEC-NN2010005	18.7	5.6	3.3
	*Megabittacus* Ren, 1997	*Megabittacus spatiosus* Yang, Ren & Shih, 2012	CNU-MEC-NN2010003	41.0	11.5	3.6
	*Formosibittacus* Li, Ren & Shih, 2008	*Formosibittacus macularis* Li, Ren & Shih, 2008	CNU-M-NN2007001	23	5	4.6
	*Jurahylobittacus* Li, Ren & Shih, 2008	*Jurahylobittacus astictus* Li, Ren & Shih, 2008	CNU-M-NN2007002	12.6	3.0	4.2
Panorpidae Latreille, 1805	*Panorpa* Linnaeus, 1758	*Panorpa kunmingensis* Fu & Hua, 2009	28-08-1985	10.0-10.3	2.1-2.5	4.1-4.8
		*Panorpa kiautai* Zhou, Hu & Wu, 1993	1982-03-25	14.0	3.0	4.7
		*Panorpa choui* Zhou, Hu & Wu, 1993	1986-07-20	14.0	3.5	4
	*Neopanorpa* Zhou, Hu & Wu, 1993	*Neopanorpa obstrusa* Zhou, Hu & Wu, 1993	1987-06-20	11.0	2.2	5
		*Neopanorpa moganshanensis* Zhou, Hu & Wu, 1993	1982-07-20	12.5	3	4.2
		*Neopanorpa tengchongensis* Zhou, Hu & Wu, 1993	1983-05-24	14	3	4.7
		*Neopanorpa menghaiensis* Zhou, Hu & Wu, 1993	1984-04-25	14.5	2.8	5.2
Choristopsychidae Martynov, 1937	*Choristopsyche* Martynov, 1937	*Choristopsyche tenuinervis* Martynov, 1937		9.5	5	1.9
			CNU-MEC-NN2011080	11.8	6.7	1.8
			CNU-MEC-NN2009317	9.5	4.9	1.9
			CNU-MEC-NN2009414	10.1	6.4	1.6
		*Choristopsyche perfecta* sp. n.	CNU-MEC-NN2009352	18.8	10.0	1.9
			CNU-MEC-NN2011082	22.2	11.4	1.9
		*Choristopsyche asticta* sp. n.	CNU-MEC-NN2009394	20.7	10.2	2.0
	*Paristopsyche* gen. n.	*Paristopsyche angelineae* sp. n.	CNU-MEC-NN2011069	11.2	7.5	1.5
			CNU-MEC-NN2011078	10.7	6.4	1.7
			CNU-MEC-NN2011076	8.4	5.5	1.5

The oval-shape forewings for choristopsychids are rather unique for mecopterans. Broad subtriangular (*Choristopsyche asticta* sp. n.) forewings can be found in *Pseudopolycentopus* (e.g. *Pseudopolycentopus janeannae* Ren, Shih & Labandeira, 2010, *Pseudopolycentopus novokshonovi* Ren, Shih & Labandeira, 2010, and *Sinopolycentropus rasnitsyni* Shih, Yang, Ren & Labandeira, 2011) with length/width ratio 2.1, 2.2 and 2.5 respectively (Ren et al. 2010c, Shih et al. 2011), slightly narrower than that of *Choristopsyche asticta* sp. n. with a subtriangular forewing and a ratio of 2.0.

Spots and bands of dark and light color are rather common for many mecopteran fossils from the Middle Jurassic of northeastern China. However, the patterns and many dark and light spots on all four wings, symmetric between left and right wings, are unique for choristopsychids (except for *Choristopsyche asticta* sp. n. without dots). It is likely that these spots and patterns on forewings might have served potential functions of mimicry, disruptive camouflage, or mate identification. The symmetric pattern between the left and right wings suggests that these dots may have been genetically controlled for an individual. We do not use the spots and bands as a diagnostic character.

The paratype of *Paristopsyche angelineae* sp. n. (CNU-MEC-NN2011078) exhibits individual asymmetry by having vein MP_3_ with two branches on the right wing, not the typical one branch on the left wing. Numerous cases of asymmetric variations within individual fossil insects have been reported from the Mesozoic of northeastern China. In the paratype of *Synapocossus sciacchitanoae* Wang, Shih & Ren, 2012 from Daohugou, China, the left and right forewings of CNU-HEM-NN2007008p/c show some individual variation, i.e., 1 mm of coalescence within the left wing and only a point contact on the right wing (Wang et al. 2012). An odonatan species, belonging to Campterophlebiidae Handlirsch, 1920, has veins MA and MP fusing before the nodus in the left wing whereas the right wing has normal venation (Zhang et al. 2008, Fig. 6). For Plecoptera, the variability of wing venation and the difference between the left and right wings of the same individual have been described in *Sinosharaperla zhaoi* Liu, Sinitshenkova & Ren, 2007 (Liu et al. 2007). *Exilibittacus lii* Yang, Ren & Shih, 2012 of Bittacidae (Mecoptera) has interesting asymmetric venational characters that RP+MA and MP of its left hind wing having only three branches and RP1+2 and MP3+MP4+CuA1+2 not forking, even though RP+MA and MP of its left and right forewings with typical four branches as those of most hangingflies (Yang et al. 2012a). Also the bittacid *Mongolbitacus daohugoensis* Petrulevičius, Huang and Ren, 2007 shows asymmetry in the anal veins of the forewings (Petrulevičius et al. 2007).

These new Chinese Choristopsychids, the first record in China, show many venational differences from the previously reported *Choristopsyche tenuinervis* Martynov, 1937. In addition, these new fossils with well preserved body structure and wings enhance our understanding of the morphological characters of this family, and provide a basis for future phylogenetic studies. Furthermore, these new species from China reveal that the early diversification of Choristopsychidae was well underway by the Middle Jurassic, while broadening the diversity of Mesozoic Mecoptera in China.

## Supplementary Material

XML Treatment for Choristopsychidae

XML Treatment for Choristopsyche

XML Treatment for Choristopsyche tenuinervis

XML Treatment for Choristopsyche perfecta

XML Treatment for Choristopsyche asticta

XML Treatment for Paristopsyche

XML Treatment for Paristopsyche angelineae
